# Crystal structure of 2-(2-methyl­phen­yl)-1,3-thia­zolo[4,5-*b*]pyridine

**DOI:** 10.1107/S2056989015012797

**Published:** 2015-07-08

**Authors:** Gamal A. El-Hiti, Keith Smith, Amany S. Hegazy, Saud A. Alanazi, Benson M. Kariuki

**Affiliations:** a Cornea Research Chair, Department of Optometry, College of Applied Medical Sciences, King Saud University, PO Box 10219, Riyadh 11433, Saudi Arabia; b School of Chemistry, Cardiff University, Main Building, Park Place, Cardiff CF10 3AT, Wales

**Keywords:** crystal structure, thi­azo­lopyridine, hydrogen bonding

## Abstract

In the title mol­ecule, C_13_H_10_N_2_S, the dihedral angle between the planes through the non-H atoms of the methylbenzene and thi­azo­lopyridine groups is 36.61 (5)°. In the crystal, the thi­azo­lopyridine groups of inversion-related mol­ecules overlap, with a minimum ring-centroid separation of 3.6721 (9) Å. Furthermore, the methylbenzene groups from neighbouring mol­ecules inter­act edge-to-face at an angle of 71.66 (5)°. In addition, weak C—H⋯ N hydrogen bonds form chains exending along [100].

## Related literature   

Various thia­zolo­pyridine derivatives have been synthesised using different synthetic methods, see: Luo *et al.* (2015 ▸); Chaban *et al.* (2013 ▸); Leysen *et al.* (1984 ▸); Lee *et al.* (2010 ▸); Rao *et al.* (2009 ▸); Johnson *et al.* (2006 ▸); El-Hiti (2003 ▸); Smith *et al.* (1994 ▸, 1995 ▸). For the X-ray crystal structures of related compounds, see: El-Hiti *et al.* (2014 ▸; 2015 ▸); Yu *et al.* (2007 ▸).
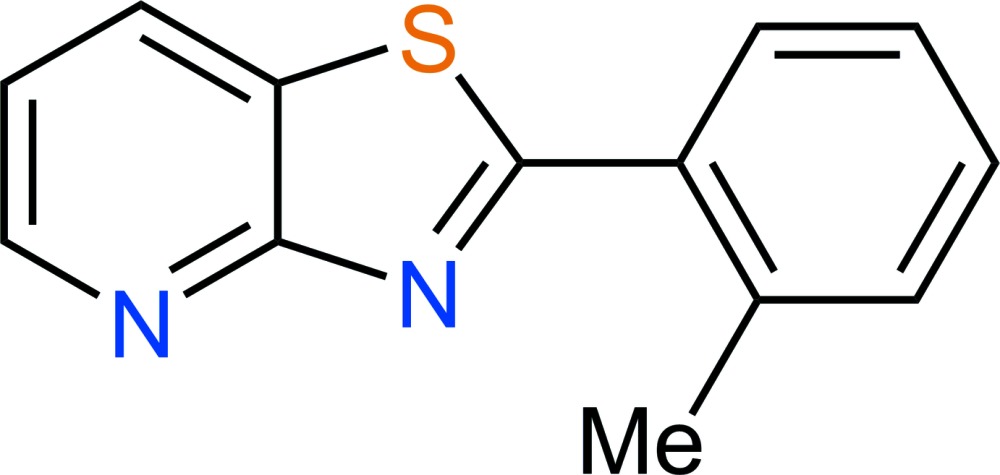


## Experimental   

### Crystal data  

C_13_H_10_N_2_S
*M* *_r_* = 226.29
Orthorhombic, 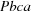

*a* = 7.6702 (1) Å
*b* = 12.6492 (3) Å
*c* = 22.9821 (5) Å
*V* = 2229.77 (8) Å^3^
*Z* = 8
Cu *K*α radiation
μ = 2.33 mm^−1^
*T* = 293 K
0.26 × 0.17 × 0.05 mm

### Data collection  

Agilent SuperNova Dual Source diffractometer with an Atlas CCD detector
Absorption correction: multi-scan (*CrysAlis PRO*; Agilent, 2014 ▸) *T*_min_ = 0.960, *T*_max_ = 0.989
7263 measured reflections
2234 independent reflections
1959 reflections with *I* > 2σ(*I*)
*R*_int_ = 0.019

### Refinement  

*R*[*F* ^2^ > 2σ(*F* ^2^)] = 0.035
*wR*(*F* ^2^) = 0.106
*S* = 1.03
2234 reflections
146 parameters
H-atom parameters constrained
Δρ_max_ = 0.17 e Å^−3^
Δρ_min_ = −0.27 e Å^−3^

### 

Data collection: *CrysAlis PRO* (Agilent, 2014 ▸); cell refinement: *CrysAlis PRO*; data reduction: *CrysAlis PRO*; program(s) used to solve structure: *SHELXS2013* (Sheldrick, 2008 ▸); program(s) used to refine structure: *SHELXL2013* (Sheldrick, 2015 ▸); molecular graphics: *ORTEP-3 for Windows* (Farrugia, 2012 ▸); software used to prepare material for publication: *WinGX* (Farrugia, 2012 ▸) and *CHEMDRAW Ultra* (Cambridge Soft, 2001 ▸).

## Supplementary Material

Crystal structure: contains datablock(s) I, New_Global_Publ_Block. DOI: 10.1107/S2056989015012797/zs2340sup1.cif

Structure factors: contains datablock(s) I. DOI: 10.1107/S2056989015012797/zs2340Isup2.hkl

Supporting information file. DOI: 10.1107/S2056989015012797/zs2340Isup3.cml

**Fig. 1.**
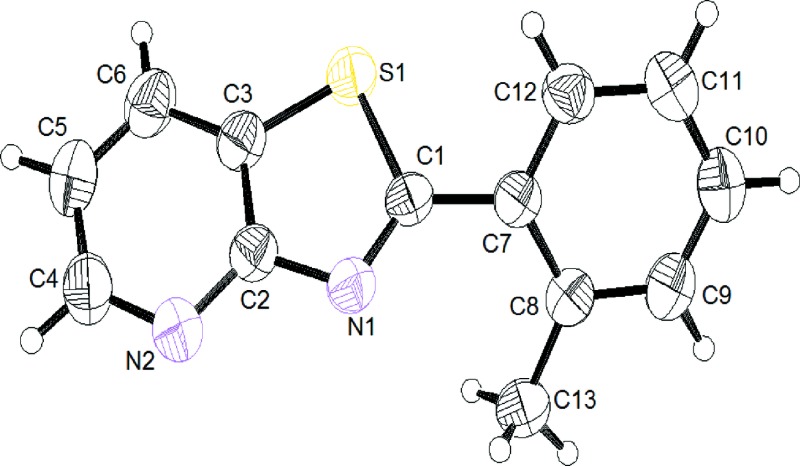
The asymmetric unit of C13H10N2O with atom labels and 50% probability displacement ellipsoids for non-hydrogen atoms.

**Fig. 2.**
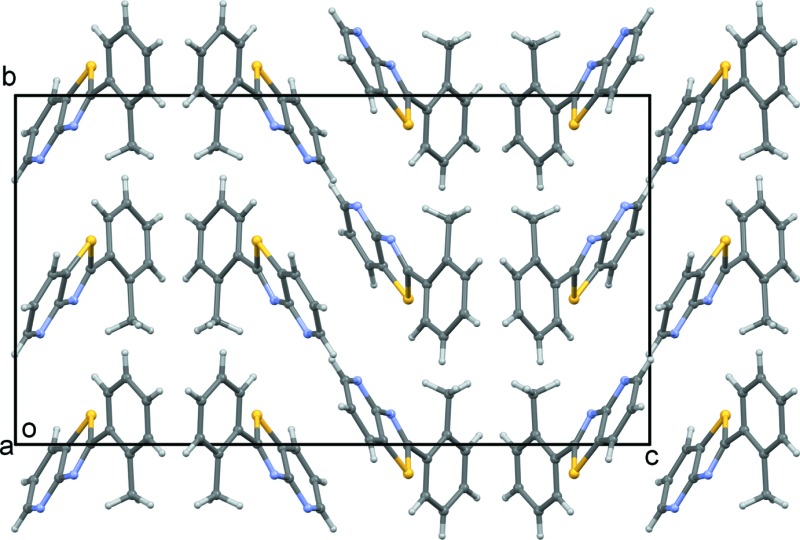
The crystal packing viewed along the a axis of the unit cell.

13 10 2 . DOI: 10.1107/S2056989015012797/zs2340fig1.tif
The asymmetric unit of C_13_H_10_N_2_O with atom labels and 50% probability displacement ellipsoids for non-hydrogen atoms.

a . DOI: 10.1107/S2056989015012797/zs2340fig2.tif
The crystal packing viewed along the *a* axis of the unit cell.

CCDC reference: 1410117

Additional supporting information:  crystallographic information; 3D view; checkCIF report

## Figures and Tables

**Table 1:** Hydrogen-bond geometry (, )

*D*H*A*	*D*H	H*A*	*D* *A*	*D*H*A*
C4H4N2^i^	0.93	2.63	3.371(2)	137

Symmetry code: (i) 
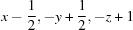
.

## supplementary crystallographic information

### S1. Introduction

Various thia­zolo­pyridine derivatives have been synthesised using different synthetic methods (Luo *et al.*, 2015; Chaban *et al.*, 2013; Leysen *et al.*, 1984; Lee *et al.*, 2010; Rao *et al.*, 2009; Johnson *et al.*, 2006; El-Hiti, 2003; Smith *et al.*, 1994, 1995). We have synthesized 2-(2-methyl­phenyl)-1,3-thia­zolo[4,5-*b*]pyridine in high yield (El-Hiti, 2003; Smith *et al.*, 1995) as a continuation of our research directed towards the development of novel synthetic routes towards heterocyclic derivatives. The X-ray structures for related compounds have been reported previously (El-Hiti *et al.*, 2014, 2015; Yu *et al.*, 2007).

### S2.1. Synthesis and crystallization

2-(2-Methyl­phenyl)-1,3-thia­zolo[4,5-*b*]pyridine was obtained in 89% yield from acid hydrolysis of 3-(diiso­propyl­amino­thio­carbonyl­thio)-2-(2-methyl­benzoyl­amino)­pyridine under reflux (Smith *et al.*, 1995) or in 61% yield from the reaction of 3-(diiso­propyl­amino­thio­carbonyl­thio)-2-amino­pyridine with 2-methyl­benzoic acid in the presence of phospho­rus oxychloride under reflux (El-Hiti, 2003). Crystallization from di­ethyl ether gave colourless crystals of the title compound. The NMR and mass spectral data for this compound were consistent with those reported (Smith *et al.*, 1995).

### S2.2. Refinement

H atoms were positioned geometrically and refined using a riding model with *U*_iso_(H) constrained to be 1.2 times *U*_eq_ for the atom it is bonded to except for methyl groups where it was 1.5 times with free rotation about the C—C bond.

### S3. Comment

The asymmetric unit consists of one molecule of C_13_H_10_N_2_S (Fig. 1). In the molecule, the angle between the least squares planes through the nonhydrogen atoms of the methyl­phenyl and thia­zolo­pyridine groups is 36.61 (5)°. In the crystal (Fig 2), the thia­zolo­pyridine groups of adjacent inversion-related molecules are parallel and overlap fully with a minimum ring centroid separation of 3.6721 (9) Å between the 5-membered and 6-membered components of the groups (related by -*x*, -*y* +1.-*z* +1) . Methyl­phenyl groups from neighbouring molecules inter­act in an edge-to-face fashion with a dihedral angle between the rings of 71.66 (5)°. A weak inter­molecular C4—H···N2^i^ contact (Table 1) forms chains of molecules extending along [100].

### Crystal data

**Table:**

C_13_H_10_N_2_S	*D*_x_ = 1.348 Mg m^−^^3^
*M**_r_* = 226.29	Cu *K*α radiation, λ = 1.54184 Å
Orthorhombic, *P**b**c**a*	Cell parameters from 3613 reflections
*a* = 7.6702 (1) Å	θ = 3.8–74.0°
*b* = 12.6492 (3) Å	µ = 2.33 mm^−^^1^
*c* = 22.9821 (5) Å	*T* = 293 K
*V* = 2229.77 (8) Å^3^	Block, colourless
*Z* = 8	0.26 × 0.17 × 0.05 mm
*F*(000) = 944	

### Data collection

**Table:**

Agilent SuperNova Dual Source diffractometer with an Atlas CCD detector	1959 reflections with *I* > 2σ(*I*)
ω scans	*R*_int_ = 0.019
Absorption correction: multi-scan (*CrysAlis PRO*; Agilent, 2014)	θ_max_ = 74.0°, θ_min_ = 3.9°
*T*_min_ = 0.960, *T*_max_ = 0.989	*h* = −9→6
7263 measured reflections	*k* = −12→15
2234 independent reflections	*l* = −28→27

### Refinement

**Table:**

Refinement on *F*^2^	0 restraints
Least-squares matrix: full	Hydrogen site location: inferred from neighbouring sites
*R*[*F*^2^ > 2σ(*F*^2^)] = 0.035	H-atom parameters constrained
*wR*(*F*^2^) = 0.106	*w* = 1/[σ^2^(*F*_o_^2^) + (0.0633*P*)^2^ + 0.2883*P*] where *P* = (*F*_o_^2^ + 2*F*_c_^2^)/3
*S* = 1.02	(Δ/σ)_max_ = 0.001
2234 reflections	Δρ_max_ = 0.17 e Å^−^^3^
146 parameters	Δρ_min_ = −0.27 e Å^−^^3^

### Special details

**Table:**

Geometry. All e.s.d.'s (except the e.s.d. in the dihedral angle between two l.s. planes) are estimated using the full covariance matrix. The cell e.s.d.'s are taken into account individually in the estimation of e.s.d.'s in distances, angles and torsion angles; correlations between e.s.d.'s in cell parameters are only used when they are defined by crystal symmetry. An approximate (isotropic) treatment of cell e.s.d.'s is used for estimating e.s.d.'s involving l.s. planes.

### Fractional atomic coordinates and isotropic or equivalent isotropic displacement parameters (Å^2^)

**Table:**

	*x*	*y*	*z*	*U*_iso_*/*U*_eq_	
C1	0.21789 (18)	0.50224 (11)	0.38130 (6)	0.0457 (3)	
C2	0.03446 (19)	0.40362 (11)	0.43105 (6)	0.0496 (3)	
C3	−0.07616 (19)	0.48902 (12)	0.42082 (6)	0.0527 (3)	
C4	−0.1768 (3)	0.31679 (15)	0.48019 (8)	0.0706 (5)	
H4	−0.2140	0.2579	0.5010	0.085*	
C5	−0.2954 (2)	0.39752 (16)	0.47141 (8)	0.0705 (5)	
H5	−0.4082	0.3917	0.4859	0.085*	
C6	−0.2461 (2)	0.48645 (16)	0.44130 (8)	0.0670 (4)	
H6	−0.3229	0.5422	0.4350	0.080*	
C7	0.37539 (18)	0.54080 (11)	0.35116 (6)	0.0470 (3)	
C8	0.48582 (19)	0.47301 (13)	0.31980 (6)	0.0527 (3)	
C9	0.6265 (2)	0.51824 (15)	0.29070 (7)	0.0644 (4)	
H9	0.6997	0.4749	0.2690	0.077*	
C10	0.6608 (2)	0.62498 (15)	0.29292 (8)	0.0675 (4)	
H10	0.7555	0.6528	0.2728	0.081*	
C11	0.5547 (2)	0.69041 (14)	0.32501 (8)	0.0664 (4)	
H11	0.5787	0.7623	0.3274	0.080*	
C12	0.4124 (2)	0.64853 (12)	0.35362 (7)	0.0563 (4)	
H12	0.3399	0.6930	0.3749	0.068*	
C13	0.4583 (3)	0.35571 (14)	0.31637 (9)	0.0730 (5)	
H13A	0.5337	0.3264	0.2872	0.109*	
H13B	0.3391	0.3414	0.3064	0.109*	
H13C	0.4845	0.3243	0.3534	0.109*	
N1	0.20074 (17)	0.41287 (9)	0.40814 (5)	0.0514 (3)	
N2	−0.0128 (2)	0.31754 (12)	0.46089 (7)	0.0659 (4)	
S1	0.03278 (5)	0.58334 (3)	0.38069 (2)	0.06317 (17)	

### Atomic displacement parameters (Å^2^)

**Table:**

	*U*^11^	*U*^22^	*U*^33^	*U*^12^	*U*^13^	*U*^23^
C1	0.0444 (7)	0.0476 (7)	0.0451 (7)	0.0040 (5)	−0.0043 (5)	−0.0027 (5)
C2	0.0486 (8)	0.0529 (8)	0.0471 (7)	0.0024 (6)	0.0008 (6)	−0.0006 (6)
C3	0.0452 (7)	0.0625 (8)	0.0502 (7)	0.0045 (6)	−0.0035 (6)	0.0007 (6)
C4	0.0698 (10)	0.0746 (11)	0.0673 (10)	−0.0082 (8)	0.0169 (8)	0.0047 (8)
C5	0.0526 (9)	0.0951 (13)	0.0637 (9)	−0.0068 (8)	0.0099 (7)	−0.0010 (9)
C6	0.0474 (8)	0.0870 (12)	0.0667 (9)	0.0106 (8)	0.0017 (7)	0.0036 (8)
C7	0.0441 (7)	0.0502 (7)	0.0468 (7)	0.0005 (6)	−0.0055 (5)	0.0007 (5)
C8	0.0497 (7)	0.0567 (8)	0.0517 (8)	0.0026 (6)	−0.0004 (6)	−0.0013 (6)
C9	0.0562 (9)	0.0778 (11)	0.0591 (9)	0.0018 (8)	0.0097 (7)	−0.0019 (8)
C10	0.0600 (9)	0.0795 (11)	0.0630 (9)	−0.0143 (8)	0.0057 (7)	0.0089 (8)
C11	0.0685 (10)	0.0609 (9)	0.0700 (10)	−0.0140 (8)	−0.0009 (8)	0.0052 (8)
C12	0.0555 (8)	0.0530 (8)	0.0605 (8)	−0.0010 (7)	−0.0038 (7)	−0.0009 (6)
C13	0.0769 (12)	0.0564 (9)	0.0856 (12)	0.0054 (8)	0.0211 (9)	−0.0104 (9)
N1	0.0490 (7)	0.0508 (7)	0.0544 (7)	0.0062 (5)	0.0033 (5)	0.0039 (5)
N2	0.0665 (8)	0.0622 (8)	0.0691 (8)	0.0026 (6)	0.0133 (7)	0.0116 (7)
S1	0.0491 (3)	0.0616 (3)	0.0789 (3)	0.01227 (16)	0.00281 (17)	0.01779 (18)

### Geometric parameters (Å, º)

**Table:**

C1—N1	1.2944 (18)	C7—C12	1.393 (2)
C1—C7	1.476 (2)	C7—C8	1.404 (2)
C1—S1	1.7518 (14)	C8—C9	1.392 (2)
C2—N2	1.337 (2)	C8—C13	1.501 (2)
C2—N1	1.3848 (19)	C9—C10	1.377 (3)
C2—C3	1.394 (2)	C9—H9	0.9300
C3—C6	1.386 (2)	C10—C11	1.375 (3)
C3—S1	1.7240 (16)	C10—H10	0.9300
C4—N2	1.334 (2)	C11—C12	1.380 (2)
C4—C5	1.382 (3)	C11—H11	0.9300
C4—H4	0.9300	C12—H12	0.9300
C5—C6	1.374 (3)	C13—H13A	0.9600
C5—H5	0.9300	C13—H13B	0.9600
C6—H6	0.9300	C13—H13C	0.9600
			
N1—C1—C7	126.56 (13)	C7—C8—C13	123.08 (14)
N1—C1—S1	115.65 (11)	C10—C9—C8	122.24 (16)
C7—C1—S1	117.79 (10)	C10—C9—H9	118.9
N2—C2—N1	120.92 (13)	C8—C9—H9	118.9
N2—C2—C3	123.54 (14)	C11—C10—C9	119.80 (15)
N1—C2—C3	115.54 (13)	C11—C10—H10	120.1
C6—C3—C2	119.80 (15)	C9—C10—H10	120.1
C6—C3—S1	130.81 (13)	C10—C11—C12	119.51 (16)
C2—C3—S1	109.37 (11)	C10—C11—H11	120.2
N2—C4—C5	124.51 (17)	C12—C11—H11	120.2
N2—C4—H4	117.7	C11—C12—C7	121.16 (16)
C5—C4—H4	117.7	C11—C12—H12	119.4
C6—C5—C4	119.85 (16)	C7—C12—H12	119.4
C6—C5—H5	120.1	C8—C13—H13A	109.5
C4—C5—H5	120.1	C8—C13—H13B	109.5
C5—C6—C3	116.67 (17)	H13A—C13—H13B	109.5
C5—C6—H6	121.7	C8—C13—H13C	109.5
C3—C6—H6	121.7	H13A—C13—H13C	109.5
C12—C7—C8	119.67 (14)	H13B—C13—H13C	109.5
C12—C7—C1	118.11 (13)	C1—N1—C2	110.40 (12)
C8—C7—C1	122.21 (13)	C4—N2—C2	115.61 (15)
C9—C8—C7	117.58 (15)	C3—S1—C1	89.04 (7)
C9—C8—C13	119.33 (14)		

### Hydrogen-bond geometry (Å, º)

**Table:**

*D*—H···*A*	*D*—H	H···*A*	*D*···*A*	*D*—H···*A*
C4—H4···N2^i^	0.93	2.63	3.371 (2)	137

Symmetry code: (i) *x*−1/2, −*y*+1/2, −*z*+1.
