# Supplementary figures and images for: Crystal structure of 2-(2-methyl­phen­yl)-1,3-thia­zolo[4,5-b]pyridine
Source: Acta Crystallogr E Crystallogr Commun. 2015 Jul 8;71(Pt 8):o562–3. doi: 10.1107/S2056989015012797 (PMC4571397; doi:10.1107/S2056989015012797)

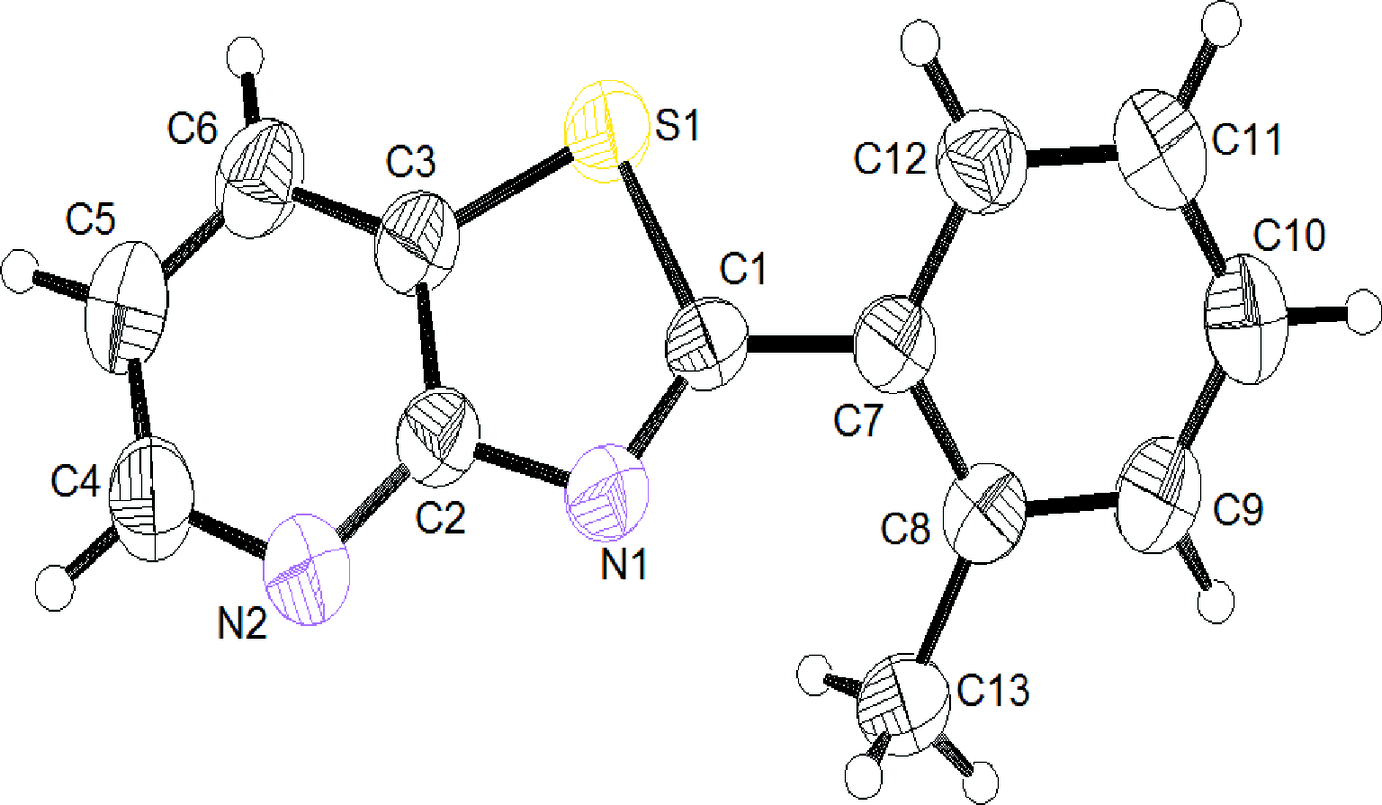

Supplement: Supplementary file 4 [file e-71-0o562-fig1.tif]

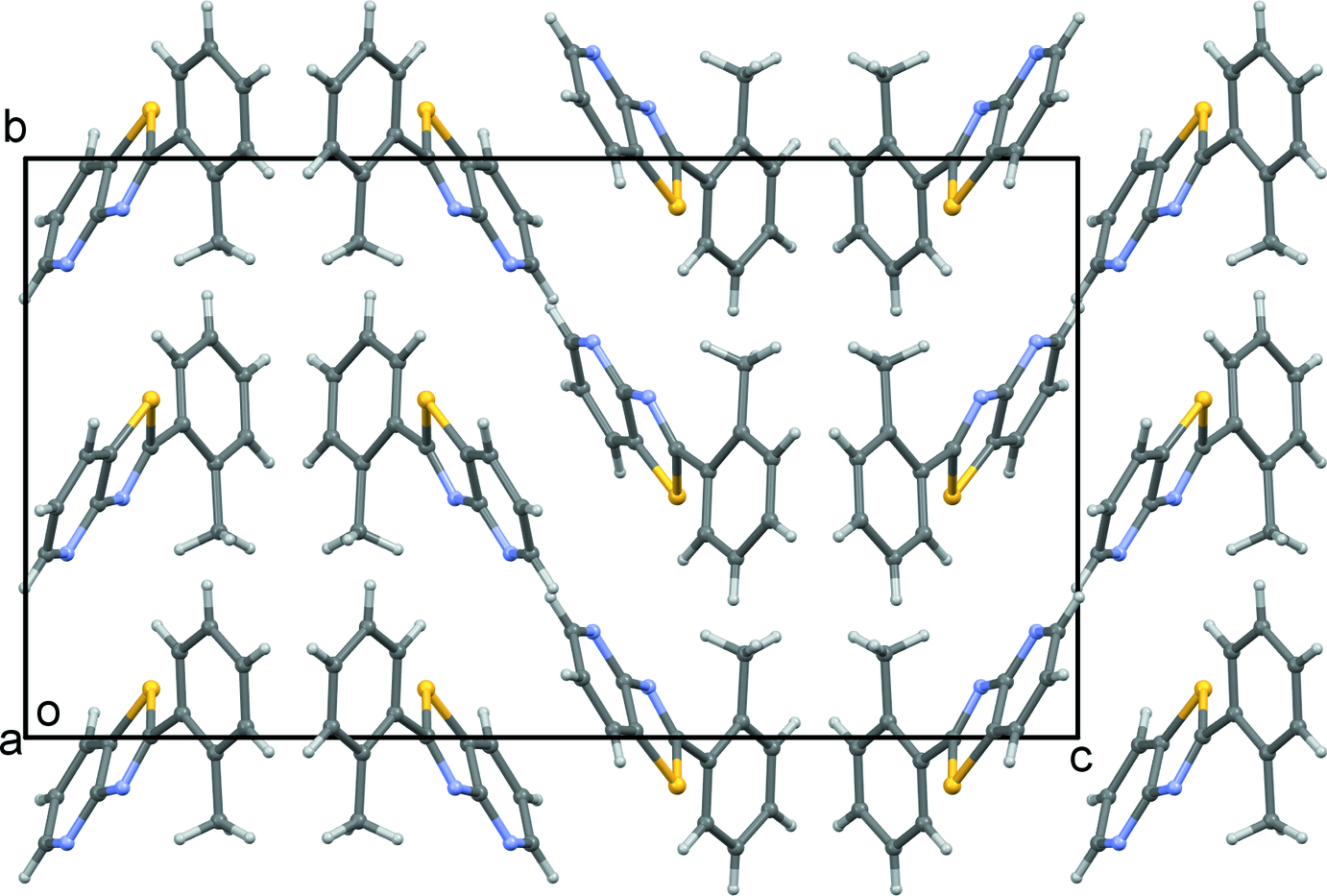

Supplement: Supplementary file 5 [file e-71-0o562-fig2.tif]
